# Coal mining as a risk factor for tuberculosis – Commodity or circumstance?

**DOI:** 10.4102/sajid.v40i1.708

**Published:** 2025-04-18

**Authors:** Nevadna Singh, Elvis M. Irusen

**Affiliations:** 1 Division of Pulmonology, Department of Medicine, Faculty of Medicine and Health Sciences, Stellenbosch University, Cape Town, South Africa

**Keywords:** coal mining, pneumoconiosis, tuberculosis, occupational disease, pulmonary disease

## Abstract

The association of tuberculosis (TB) with the mining industry is well known in South Africa. This may well be true for gold mining and silica exposure. However, a description of the pathophysiological link between coal mining and TB is not clearly detailed in the literature with many articles citing associations rather than direct causality. A focused intervention of active case-finding and treatment in coal mines in South Africa resulted in a dramatic reduction in the incidence of TB of more than 70% compared to historical figures and substantially below the national incidence as well.

**Contribution:**

We suggest that the risk of acquiring or reactivating TB is not solely attributable to coal exposure itself. Instead, socioeconomic factors, such as poor working and living conditions around mines and comorbid illnesses, likely play a more significant role, as the principal drivers of the disease and therefore, these factors, alongside active screening for TB should receive more attention.

## Introduction

South Africa, a leading coal mining country, has long been associated with a higher prevalence of tuberculosis (TB) among miners. This relationship is recognised in various occupational health regulations, such as the *Occupational Diseases in Mines and Works Act of 1973* (ODMWA), which includes TB among compensable conditions for miners.^1^ Historically, TB has disproportionately affected the mining sector in sub-Saharan Africa, where socioeconomic factors often exacerbate disease risk. Poor working and living conditions, migrant labour, over-crowding, co-morbid illnesses and malnutrition are root risk factors for susceptibility to TB, all of which are prevalent in the South African coal mining industry. This article highlights discrepancies concerning coal as an isolated aetiological agent in the development of pulmonary TB, balancing its relative significance against other socioeconomic factors as drivers of the disease.

### Historical context and pathophysiology

The impact of silica exposure on lung health, including its role in TB susceptibility, has been known since the late 19th century. The depressant effect of crystalline silica on the ability of alveolar macrophages to kill *Mycobacterium tuberculosis (M.tb)* was later confirmed in experimental studies.^2^ Research indicates that silica exposure alone, even in the absence of silicosis, raises the lifelong risk of TB.^3,4^

In contrast, the relationship between coal dust exposure and TB is less clear. Dust exposure in coal mines is an established risk factor for occupational lung diseases such as coal workers’ pneumoconiosis (CWP) and chronic obstructive pulmonary disease (COPD).^5^ Unlike silicosis, the direct evidence, and a causal relationship between coal dust exposure with or without CWP and TB is, in fact, tenuous. There is plausible doubt about coal dust exposure as a direct risk for *M.tb* infection. Pathophysiological mechanisms explaining this association are lacking. Ross, of the South African Safety in Mines Research Advisory Committee, has stated that ‘CWP alone does not carry an increased risk for mycobacterial infection, either by *M.tb* or nontuberculous Mycobacteria (NTM)’.^3^ The current understanding of TB pathogenesis has suggested that adult TB cases are linked to either primary infection or the reactivation of latent TB acquired during childhood or adolescence. Both pathways are significant contributors to developing active TB in adults, underscoring the complex nature of its progression and the various factors influencing disease manifestation. It is noteworthy that the Centre for Disease Control and Prevention (CDC), in a comprehensive review of potential sequelae of coal dust exposure, does not list TB as a complication, but it must be noticed that these data mainly originates from the United States of America.^6,7^

### Socioeconomic and environmental factors

The first comprehensive study of South African coal miners in 1963 highlighted a significant prevalence of both TB (25.4%) and CWP (26.8%) among 1010 coal miners who underwent autopsy.^8^ Since then, various studies have suggested a causal link between coal mining and TB, including positive correlations with CWP and dose dependent relationships with coal dust exposure.^9,10,11^ Importantly, silica may be a component of coal dust, although not always, and is present in varying degrees, depending on coal rank. Substantial concern exists regarding the reliability of silica measurements in both the mining and non-mining sectors, as sampling errors, equipment calibration, environmental factors and competing particle interference can all influence readings.^12^ Despite inaccuracies in absolute measurements of silica content, this concept of variability is highly relevant when interpreting causality, as higher silica content in higher-rank coal may exacerbate the risk of mixed dust pneumoconiosis, silicosis and TB.^2^ Geographical variability in coal rank and silica content is wide, with South African coal mines falling into lower ranks with less silica content.^2^

More recent South African research, spanning over 20 years, has revealed a paradox: white coal miners, who generally had longer exposure durations than their black counterparts, exhibited a lower TB incidence. It was found that black coal miners had an 8.3-fold greater risk for TB than white coal miners (*p* < 0.0001) despite the mean duration of coal dust exposure of 8 years versus 21 years, respectively. This discrepancy likely reflects broader socioeconomic disparities rather than a direct effect of coal exposure. Factors such as income, nutrition, living conditions, and access to medical care differed significantly between black and white miners, with race often serving as a proxy for these socioeconomic inequalities. Additionally at the time, migrant labour and inconsistent healthcare access likely contributed to higher TB rates among black miners.^13,14^ Whether the same type of mining work activities was performed by black and white coal miners was not reported in this autopsy study. From the late 1990s, human immunodeficiency virus (HIV) would increasingly change the medical landscape to be the leading driver of TB in South Africa, particularly on the mines, where unsafe sexual behaviour was common. These factors synergistically constitute the primary reasons for TB reactivation and subsequent spread of infection among people in close contact, living and working around the mines and underground.

### Public health interventions and findings

Recent data from 2014 to 2022 demonstrate that public health initiatives, including proactive TB case finding and treatment, significantly reduced TB incidence by over 70% among miners.^15^ The Minerals Council reported that the TB incidence rate dropped from 1060/100 000 in 2014 to 278/100 000 in 2022, dipping well below the national community incidence rate (537/100 000).^15^ The magnitude of this decline suggests that the reduction in TB cases is more attributable to effective public health measures than to changes in coal exposure alone.

A 2020 study in Malawi found that miners in the coal industry had a lower likelihood of pulmonary TB than those in quarry or other types of mining (OR = 0.30, 95% CI:0.19–0.49, *p* < 0.001).^14^ This may support the notion that coal mining itself may not be a direct risk factor for TB, although this study did not fully explore the exact nature of the exposures. It seems that it is the socio-economic conditions of congested residential living around the mines and poor ventilation in crowded, underground mining that promote transmission of TB and the development of disease.

## Conclusion

Our current review of the evidence suggests that a direct correlation and causal relationship between coal dust exposure or CWP and TB may be lacking. Effective public health interventions have demonstrated significant success in reducing TB incidence among miners. Future efforts should continue to address socioeconomic determinants, improve living conditions, enhance nutrition, HIV prevention and control, and ensure equitable access to healthcare. The hypothesis that coal mining is an independent risk factor for TB should therefore be reconsidered, focusing instead on the broader context of health disparities and living conditions.
